# Exploring systemic erythropoietin in diabetic retinopathy: Longitudinal associations and biomarker potential in the LANDMark cohort

**DOI:** 10.1371/journal.pone.0358455

**Published:** 2026-09-15

**Authors:** Ingrid A. Wise, Siska Dupont Berry, Alisa McKenzie, Jason C. Steel, Katie Edwards, Nathan Efron, Christopher J. Layton, Charmaine A. Ramlogan-Steel

**Affiliations:** 1 School of Health, Medical and Applied Sciences, Central Queensland University, Cairns QLD, Australia; 2 School of Health, Medical and Applied Sciences, Central Queensland University, North Rockhampton, QLD, Australia; 3 Centre for Vision and Eye Research, School of Clinical Sciences, Queensland University of Technology, Brisbane, QLD, Australia; 4 Faculty of Medicine, Greenslopes Clinical School, The University of Queensland, Brisbane, QLD, Australia; 5 LVF Ophthalmology Research Centre, Translational Research Institute, Brisbane, QLD, Australia; Kamuzu University of Health Sciences, MALAWI

## Abstract

**Aims:**

Diabetes mellitus (DM) is a chronic, multifactorial disease with systemic effects, demanding ongoing clinical vigilance and risk-reduction strategies. Among its most prevalent microvascular complications is diabetic retinopathy (DR), affecting 30–40% of individuals with DM and associated with sustained hyperglycaemia. This study investigates the role of erythropoietin (EPO), a glycoprotein primarily known for its role in erythropoiesis, in retinal pathology.

**Methods:**

Secondary analysis was conducted on 117 participants from the Australian cohort of the LANDMark study, examining the relationship between systemic EPO levels and DR severity, as measured by the Early Treatment Diabetic Retinopathy Study (ETDRS) Severity Scale.

**Results:**

Significant positive correlations were observed between serum EPO and DR severity at both baseline and the four-year follow-up (baseline: ρ = 0.212, *p* = 0.025; four-year ρ = 0.205, *p* = 0.030). Ordinal regression indicated that both baseline and four-year EPO levels were associated with higher DR severity at the four-year follow-up (baseline: log-odds coefficient = 2.258, OR ≈ 9.57, *p* < 0.01; four-year: log-odds coefficient = 2.111, OR ≈ 8.26, *p* = 0.01).

**Conclusion:**

These findings suggest that systemic EPO shows a modest association with subsequent retinopathy severity and may reflect an exploratory biomarker of ocular disease activity.

## Introduction

Diabetes mellitus (DM) is a progressive and multifaceted condition that affects multiple organ systems and necessitates lifelong clinical management [1]. In 2022, the global prevalence of DM was estimated at 828 million adults, up from 630 million in 1990 [2]. The increased prevalence of DM, particularly type 2 diabetes mellitus (T2DM), which accounts for approximately 90% of all cases, is largely attributed to aging populations, obesogenic environments, and rapid urbanisation [3]. In contrast, the incidence of type 1 diabetes mellitus (T1DM) is also rising, but the underlying drivers of this trend are less clear [3].

The burden of DM extends beyond metabolic dysregulation, with outcomes such as amputation, renal failure, and vision loss contributing to significant morbidity and mortality. Diabetic retinopathy (DR), a leading cause of blindness among working age adults, exemplifies the vascular and neurodegenerative consequences of chronic hyperglycaemia [4]. The rising prevalence of DM among younger individuals is particularly concerning, as their longer lifetime exposure to the disease increases the risk of developing associated complications [5].

Traditionally, DM complications are classified as either macrovascular or microvascular, with only a small subset of conditions grounded in both aetiologies [5]. Global observational studies, such as the DISCOVER study, have reported that approximately 12.7% of patients with DM present with macrovascular complications, while 18.8% present with microvascular complications [6]. Despite their effectiveness in reducing microvascular complications, traditional glucose lowering strategies aimed at risk reduction have not demonstrated significant benefits in lowering overall mortality or macrovascular complications, as demonstrated in the UK Prospective Diabetes Study Group (UKPDS) trials [7,8]. These findings are reinforced by other studies, which demonstrate that DM management strategies centred exclusively on glycaemic control are insufficient for reducing complications rates or overall mortality [9,10].

Visual impairment in DR arises from two primary mechanisms – neovascularisation in proliferative DR and vascular leakage in diabetic macular oedema [11]. Both conditions are influenced by hypoxia induced angiogenic factors, notably vascular endothelial growth factor (VEGF) [11,12]. The adoption of DR classification systems, such as the Early Treatment of Diabetic Retinopathy Study (ETDRS) diabetic retinopathy severity scale (DRSS), has effectively helped quantify the risk of disease progression [13]. In addition to retinal grading systems, ophthalmic imaging techniques offer complementary insights into diabetic complications. Corneal confocal microscopy (CCM), a non-invasive method for assessing corneal nerve morphology, has shown promise as a surrogate marker for peripheral neuropathy and systemic neurodegeneration in DM [14]. Given the shared microvascular and neurodegenerative pathways between diabetic neuropathy and retinopathy, CCM-derived metrics may provide valuable context for understanding retinal disease progression.

However, despite these advancements, significant gaps remain in the prevention and management of DR, and it continues to be a common complication of DM and a leading cause of preventable blindness in the adult working population [15]. One notable gap in current understanding relates to erythropoietin (EPO), a hypoxia-responsive cytokine that has been shown to exert both neuroprotective and angiogenic effects in the retina [16].

Given its dual capacity for neuroprotection and angiogenesis, EPO represents a paradoxical factor in DR: potentially protective in early stages, yet pathogenic in proliferative disease [16–18]. In the eye, local EPO production and expression of the erythropoietin receptor (EPOR) has been identified in multiple retinal layers, supporting both protective and pathogenic roles depending on the disease stage [19,20]. Consequently, therapeutic strategies aimed at suppressing EPO to mitigate neovascularisation must carefully balance its neuroprotective functions [21].

Although intraocular EPO has been studied in the context of neovascularisation, the influence and prognostic value of circulating (systemic) EPO for DR severity and progression remains uncertain. Prior work has been largely cross-sectional, limiting conclusions about causality or temporal dynamics. It remains uncertain whether circulating EPO acts partially to curtail established retinal damage, exacerbates the diabetic process in the retina, or serves as an early biomarker of disease progression. Longitudinal data are essential to clarify this distinction and to assess whether systemic EPO is merely a correlate of established disease or a true predictor and possibly contributor to future progression. Addressing these complexities is essential for reducing the burden of vision loss and improving outcomes for individuals with DM.

The Longitudinal Assessment of Neuropathy in Diabetes using novel ophthalmic Markers (LANDMark) study is a prospective observational study involving individuals with T1DM, T2DM, and non-diabetic controls. It was originally designed to evaluate corneal nerve morphology and function as indicators of peripheral diabetic neuropathy [14,22]. However, the LANDMark dataset also provides valuable insights into DR, offering an opportunity to explore the role of EPO in retinal pathology. By leveraging longitudinal follow-up, these analyses aim to clarify the relationship between systemic EPO levels and DR, addressing this important knowledge gap and informing the potential utility of EPO as a biomarker for risk stratification and therapeutic target in diabetic eye disease.

## Methods

LANDMark is a five-year observational study conducted at the Anterior Eye Laboratory, Institute of Health and Biomedical Innovation, Queensland University of Technology, Australia and the Division of Cardiovascular Medicine, University of Manchester, United Kingdom. The written consent process and recruitment period for this study are as previous described and published [14,22]. In LANDMark, all participants, regardless of diabetes status, underwent detailed neuropathy and corneal nerve assessments at 12-month intervals over a five-year period. Similarly, general health examinations were conducted at each visit, including medical history questionnaires, measurements of glycaemic control (glycated haemoglobin A1c [HbA1c]), renal function (albumin-to-creatinine ratio [ACR]) and lipid profiles. Participants had no previous history of eye surgery, injury, disease, or systemic illnesses (except diabetes) that may have influenced the cornea. Similarly, with the exception of DM, participants had no other identified causes of peripheral neuropathy. Baseline and four-year subject demographics are reported in Table 1. This portion of the study was approved by the Queensland University of Technology, the Princess Alexandra Hospital, Brisbane, and the Mater Health Services Human Research Ethics Committees, and was conducted in accordance with the tenets of the Declaration of Helsinki.

**Table 1:** Characteristics and clinical measures of study participants. Values shown are mean ± standard deviation with the standard error in parenthesis. or counts for categorical variables.

Characteristics	At Baseline			At 4-Years		
	Non-diabetic	Diabetic	*P* value	Non-diabetic	Diabetic	*P* value
	Mean ± SD (SE)	Mean ± SD (SE)		Mean ± SD (SE)	Mean ± SD (SE)	
Height(cm)	170.50 ± 8.71 (1.31)	171.55 ± 10.53 (1.24)	N/S	170.29 ± 8.74 (1.32)	171.22 ± 10/46 (1.24)	N/S
Weight(kg)	76.70 ± 14.36 (2.16)	80.96 ± 16.93 (2.00)	N/S	76.75 ± 13.09 (1.97)	81.31 ± 17.17 (2.04)	N/S
Waist circumference(cm)	89.29 ± 12.07 (1.88)	91.92 ± 15.07 (1.85)	N/S	89.56 ± 9.82 (1.48)	93.74 ± 14.64 (1.74)	<0.05
BMI (kg/m^2^)	26.33 ± 4.29 (0.65)	27.48 ± 5.36 (0.63)	N/S	26.43 ± 3.88 (0.59)	27.71 ± 5.39 (0.64)	N/S
HbA1c (mmol/mol)	35.37 ± 3.7 (5.5)	63.0 ± 14.2 (1.8)	<0.001	32.5 ± 5.6 (0.9)	64.3 ± 19.2 (2.3)	<0.001
Total cholesterol (mmol/L)	5.47 ± 1.21 (0.18)	4.61 ± 0.89 (0.10)	N/S	5.40 ± 1.13 (0.17)	4.65 ± 1.08 (0.13)	<0.001
Triglycerides(mmol/L)	1.08 ± 0.59 (0.98)	1.10 ± 0.69 (0.08)	N/S	1.13 ± 0.52 (0.08)	1.20 ± 0.90 (0.11)	N/S
HDL cholesterol (mmol/L)	1.48 ± 0.38 (0.06)	1.54 ± 0.51 (0.06)	N/S	1.40 ± 0.34 (0.05)	1.46 ± 0.38 (0.04)	N/S
LDL cholesterol (mmol/L)	3.72 ± 1.92 (0.29)	2.62 ± 0.74 (0.09)	N/S	3.47 ± 1.06 (0.16)	2.64 ± 0.89 (0.09)	N/S
Supine Systolic blood pressure (mmHg)	117.29 ± 13.79 (20.8)	120.53 ± 17.18 (2.02)	N/S	116.00 ± 12.65 (1.91)	119.84 ± 14.03 (1.67)	N/S
Supine Diastolic blood pressure(mmHg)	73.18 ± 6.85 (1.03)	71.94 ± 8.71 (1.03)	N/S	71.40 ± 8.05 (1.21)	72.03 ± 7.18 (0.85)	N/S
Urine albumin (mg/L)	8.91 ± 25.47 (3.98)	20.94 ± 52.12 (6.28)	<0.05	7.39 ± 10.82 (1.65)	19.23 ± 49.32 (5.94)	<0.05
Urine alb/creat ratio (mg/mmol)	1.03 ± 1.70 (0.26)	4.37 ± 14.96 (1.83)	<0.05	1.29 ± 1.63 (0.25)	2.99 ± 8.12 (0.98)	<0.05
Creatinine (umol/L)	75.43 ± 13.84 (2.11)	75.063 ± 19.73 (2.36)	N/S	74.33 ± 12.10 (1.82)	82.61 ± 21.83 (2.59)	N/S
eGFR (mL/min)	79.48 ± 11.41 (1.74)	79.91 ± 16.41 (2.02)	N/S	84.40 ± 8.27 (1.25)	82.14 ± 11.88 (1.43)	N/S

BMI, body mass index; HbA1c, glycated haemoglobin; HDL, high-density lipoprotein; LDL, low-density lipoprotein; eGFR, estimated glomerular filtration rate.

This study reports on 117 participants from the Australian cohort of the LANDMark study for whom serum samples were available [22], which consisted of 72 individuals with T1DM and 45 healthy, non-diabetic controls. The deidentified data was accessed for this investigation from 29 November 2024–30 August 2025. It builds on the work by Andrzejewski et al. [23], which analysed serum samples from LANDMark participants. The serum samples analysed were collected from each participant at two different timepoints, approximately four years apart. Blood collection was performed after at least 12-hour fasting and sera samples stored at −80°C until analysis. Exclusion criteria are as previously described [23]. Ethics approval for this further analysis was obtained from the Queensland University of Technology and the Greenslopes Private Hospital Ethics Committee. Sera were analysed at Greenslopes Hospital, Brisbane. Written informed consent was obtained from all participants.

### Primary outcome

The primary outcome of this study was DR severity at the four‑year follow‑up, measured using the ETDRS Severity Scale. Ordinal logistic regression models treated four‑year ETDRS severity as the dependent outcome. Longitudinal changes in ETDRS scores between baseline and four years were examined descriptively to characterise disease trajectory; however, the study was not designed to model formal progression endpoints.

### Regression modelling and covariate selection

Ordinal logistic regression models were used to examine associations between systemic EPO levels and DR severity at the four‑year follow‑up, measured using the ETDRS Severity Scale. Unadjusted models evaluated the association between EPO and four‑year DR severity, followed by multivariable models incorporating clinically relevant covariates.

Covariates included in adjusted models were prespecified in the original LANDMark study protocol and selected for this secondary analysis based on established clinical relevance to DR. These included age, HbA1c, and ACR, reflecting cumulative disease exposure, glycaemic burden, and microvascular disease, respectively. Additional systemic variables measured in LANDMark (lipid profiles, blood pressure) were not included in multivariable models to reduce overfitting and because they were not central to the primary hypothesis of this analysis, given the sample size. Multicollinearity was assessed using variance inflation factors (VIFs). Model assumptions for ordinal regression were assessed and considered acceptable.

### Data analysis

All statistics analyses were performed using SPSS version 29 [24] and R [25]. Independent t-tests and Fisher’s exact tests were used to assess statistical significance between variables. The results were analysed to provide rationale for including or excluding each variable as a covariate in further analyses (Table 1). Nonparametric tests, including Mann-Whitney U and Spearman’s correlation analyses, were used for variables that did not meet assumptions of normality, while analyses such as linear and ordinal regression modelling were applied as appropriate. Missing data were addressed using multiple imputation (MI). The MI method utilised 10 imputations, calculated and applied using the Multivariate Imputation by Chained Equations (MICE) procedure with Predictive Mean Matching (PMM), as outlined by Heymans and Twisk [26]. Clinical data reported in below-threshold or above-threshold formats were adjusted to the respective threshold values for statistical analysis [26]. For example, values reported as <0.2 (units) were recorded as 0.2 (units), enabling the data to be treated as continuous and allowing for appropriate statistical analysis (S1-S3 Tables).

### Missing data and sensitivity analyses

Missing data in the original LANDMark dataset were addressed using multiple imputation, with the extent and distribution of pre‑imputation missingness summarised in S2 and S3 Tables. To assess whether imputed values influenced the findings, sensitivity analyses were undertaken by (i) repeating the primary ordinal regression models using only participants with complete baseline data prior to imputation and (ii) including an indicator variable representing pre‑imputation completeness in the adjusted regression models.

## Results

### Participant characteristics

A subset of 117 participants (45 controls and 72 individuals with T1DM) were included in this study from the greater LANDMark study cohort. Table 1 presents the control and T1DM population characteristics at baseline and four-years. As expected, baseline T1DM participants had significantly elevated HbA1c (*p* < 0.001), urine albumin *(p* < 0.05)*,* and urine ACR *(p* < 0.05), compared to their non-diabetic counterparts. This difference reflected typical underlying pathology associated with T1DM and significantly distinguished the two cohorts. These differences persisted at the four-year follow up, in addition to significant between-group differences for waist circumference (*p* < 0.05) and total cholesterol (*p* < 0.001). The control and T1DM participants were well-matched for gender, weight, and blood pressure and therefore analyses were not adjusted for these matched cofactors. These trends affirmed the sample population as representative of the broader LANDMark cohort.

### Confocal microscopy

Outcomes of CCM assessment of this subset of patients are shown in S1 Fig. Corneal nerve fibre density (CNFD), branch density (CNBD), and fibre length (CNFL) are well-established CCM markers of peripheral neurodegeneration [27]. A significant *(p <* 0.001) decrease of corneal nerve fibre density (CNFD), branch density (CNBD), and fibre length (CNFL) between T1DM and control subjects was seen at both timepoints. The trends align with previous publications of the broader LANDMark cohort data, demonstrating data reliability and validity.

### Diabetic retinopathy severity

There was a statistically significant difference in ETDRS DRSS between the control and the diabetes group at both baseline (*p* < 0.001) and four-years (*p* < 0.001). Individuals with T1DM experienced a significant increase in ETDRS DRSS scores over the four-year period, as shown in S2 Fig (t = −4.472, P < 0.001, CI [−0.1976, −0.067]), indicating progression of DR. Among the T1DM participants, 39.4% showed increased ETDRS DRSS scores, reflecting worsening retinal pathology; 56.1% remained stable, and 4.5% demonstrated improvement. In contrast and as expected, the control group did not exhibit any significant change in ETDRS DRSS (t = −1.849, p > 0.05, CI [−0.144, 0.006]), suggesting stable retinal status with regards to the ETDRS markers over time. These findings are consistent with the existing literature and reinforce the generally progressive nation of DR in individuals with DM.

### EPO and markers of diabetic retinopathy

#### EPO difference between cohorts.

**Fig 1:**
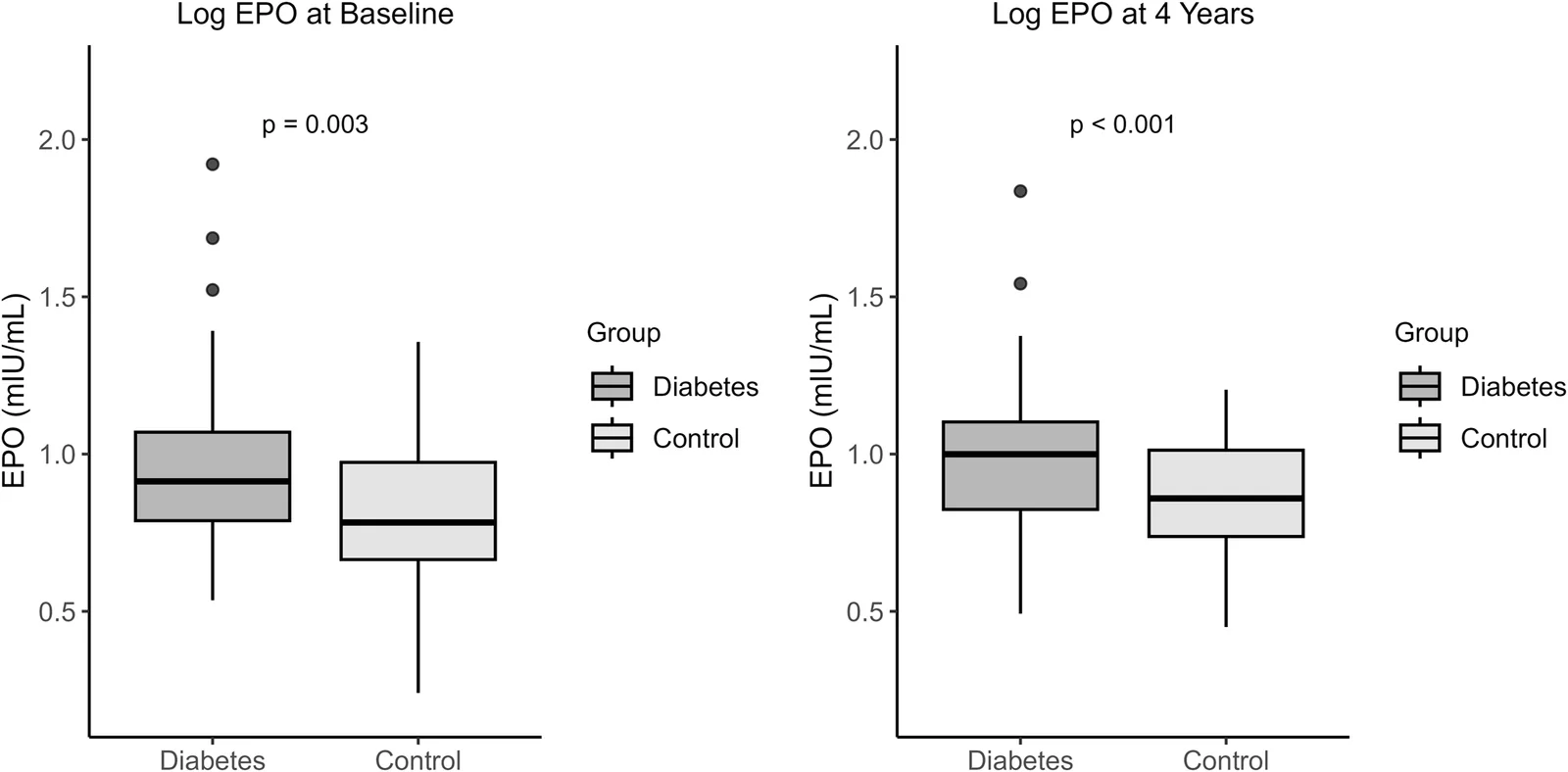
Baseline EPO. Mann-Whitney U tests revealed significant differences between the control and diabetes groups for EPO levels at both baseline (U = 1051.500, Z = −3.185, p = 0.003) and 4-years (U = 1047.000, Z = −3.210, p < 0.001), with the diabetes group exhibiting higher EPO levels than the control group at both time points.

EPO was significantly elevated among the T1DM cohort, both at baseline (Z = −3.185, p = 0.003) and four-years (Z = −3.210, p < 0.001), compared to the non-diabetic controls (Fig 1).

#### EPO and structural ocular changes in diabetes.

Spearman’s rank correlation was used to assess the strength and direction of any relationship between EPO and the CCM measures of corneal neuropathy, namely CNFD, CNBD and CNFL. Whilst the corneal nerve parameters are strongly correlated with each other at the 4-year follow-up, EPO levels did not show a significant correlation with any of the corneal nerve parameters at baseline or four-years.

#### EPO and ETDRS DRSS score.

**Fig 2:**
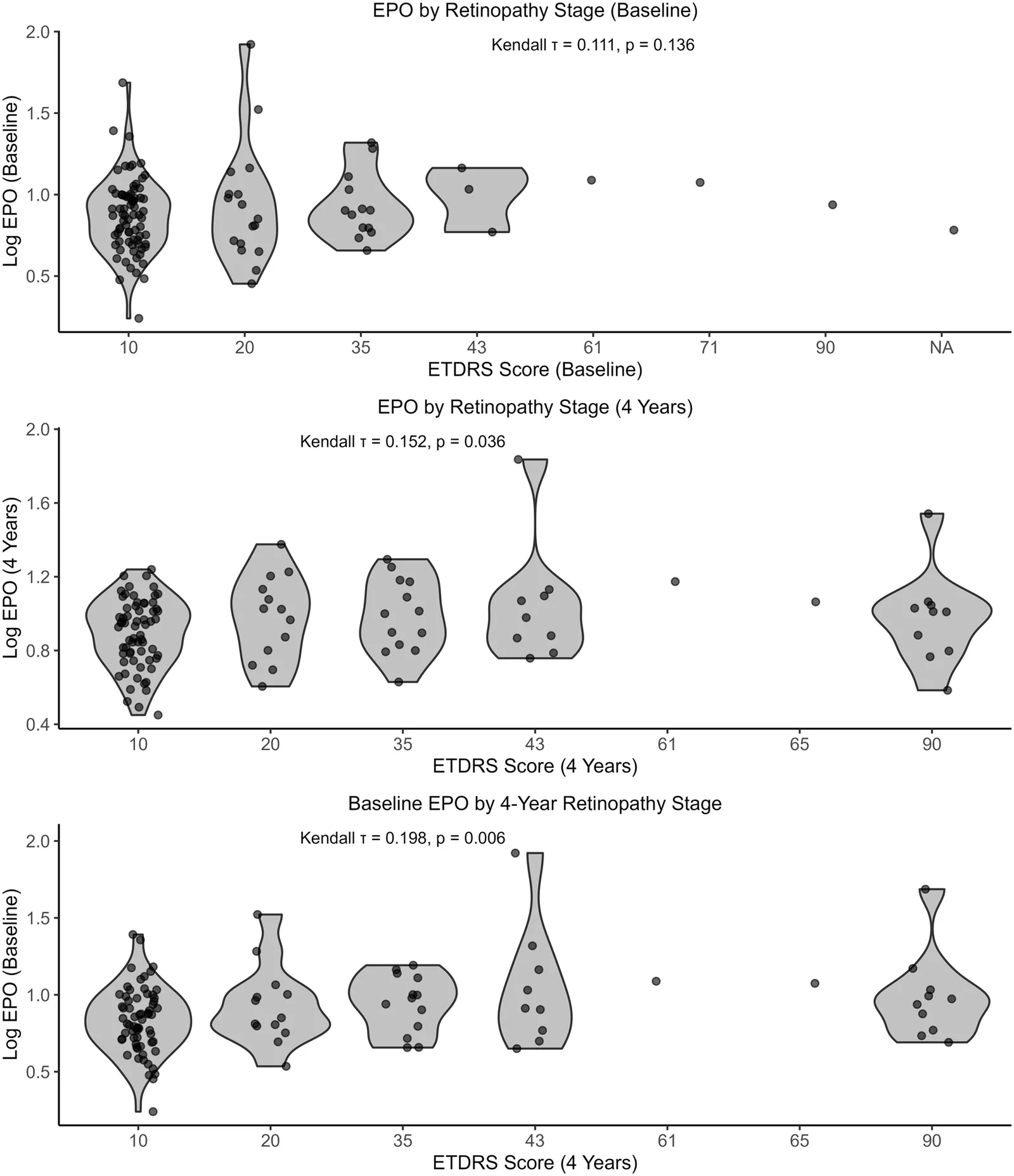
Relationship between EPO and ETDRS DRSS. Kendall’s tau-b analyses were conducted to examine the relationship between EPO and ETDRS DRSS scores at baseline and four years. At baseline, non-significant positive association was observed between baseline EPO and ETDRS DRSS (τ = 0.111, p = 0.136). At four years, a weak but statistically significant positive association was observed between 4-year EPO and 4-year ETDRS DRSS (τ = 0.152, p = 0.036). Similarly, baseline EPO was also modestly, but significantly, associated with ETDRS DRSS at four years (τ = 0.198, p = 0.006).

Kendall’s tau-b analyses were conducted to examine the relationship between EPO levels and ETDRS DRSS scores at baseline and four years (Fig 2). At baseline, there was a weak, non-significant positive association between baseline EPO and baseline ETDRS DRSS (τ = 0.111, p = 0.136), suggesting a trend toward higher EPO with greater DR severity, although this did not reach statistical significance. At four years, EPO measured concurrently with ETDRS showed a weak but statistically significant positive association (τ = 0.152, p = 0.036), indicating that higher EPO levels were modestly associated with more severe retinopathy at follow-up. Additionally, baseline EPO was weakly but significantly associated with ETDRS DRSS measured at four years (τ = 0.198, p = 0.006), suggesting that elevated EPO early in the disease course may predict subsequent DR severity. Kendall’s tau is a non-parametric measure of monotonic association appropriate for ordinal outcomes; however, it does not account for potential confounders or allow inference on independent effects. Therefore, these findings are descriptive and should be interpreted in the context of subsequent ordinal regression analyses. Moreover, the magnitude of these associations was small, indicating modest effect sizes that support exploratory interpretation rather than clinical prediction.

#### EPO Regression analysis.

**Fig 3:**
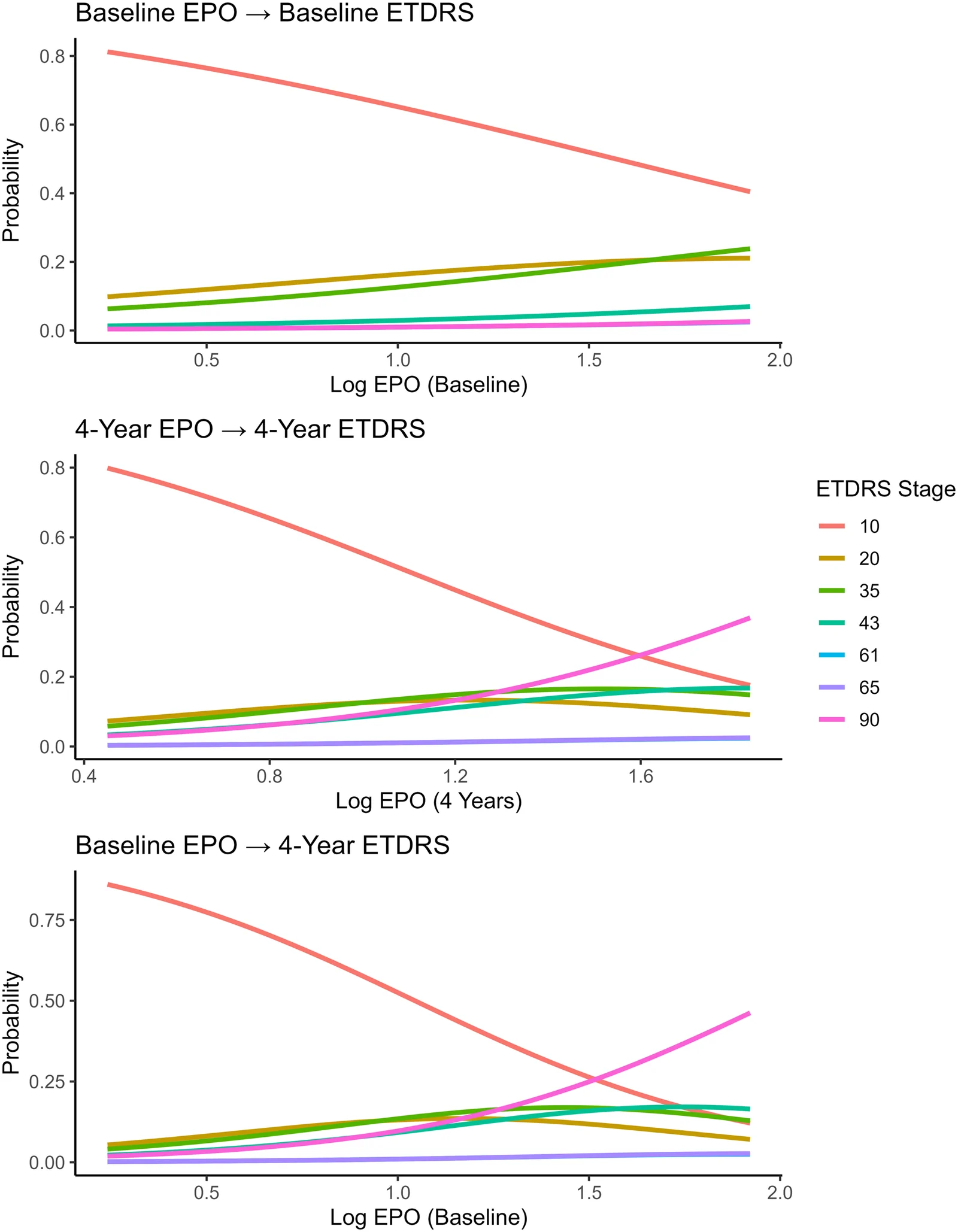
EPO prediction probability. Predicted probabilities of ETDRS retinopathy score by systemic EPO levels. Ordinal logistic regression models were used to estimate the likelihood of being in each ETDRS score based on log-transformed EPO. Top panel: Baseline EPO predicting baseline ETDRS score (log-odds coefficient = 1.101, OR ≈ 3.01). Middle panel: EPO at 4 years predicting ETDRS score at 4 years (log-odds coefficient = 2.111, OR ≈ 8.26). Bottom panel: Baseline EPO predicting ETDRS score at 4 years (log-odds coefficient = 2.258, OR ≈ 9.57). Each coloured line represents an ETDRS score, with higher EPO levels associated with increased probability of more severe retinopathy. Thresholds are included in the model to define the cumulative logit between stages.

Ordinal logistic regression analyses demonstrated that both baseline and four-year systemic EPO levels were significantly associated with DR severity in unadjusted models at the four-year follow-up, as assessed by the ETDRS DRSS (Fig 3). EPO values were log-transformed for the analyses, but values are presented in the original units (mIU/mL) for interpretability. Baseline EPO was positively associated with four-year DR severity (log-odds coefficient = 2.258, OR ≈ 9.57), indicating that a 1-unit increase in log-transformed baseline EPO, corresponding to an approximate 2.7-fold increase in actual EPO, was associated with nearly tenfold higher odds of being in a more severe ETDRS score. Four-year EPO was similarly associated with concurrent DR severity (log-odds coefficient = 2.111, OR ≈ 8.26), with a 1-unit increase in log-transformed EPO corresponding to an approximate 2.7-fold increase in EPO and an eightfold increase in the odds of a higher ETDRS score. These results demonstrate that elevated systemic EPO, both at baseline and at four years, is strongly associated with increased DR severity.

#### ETDRS DRSS Multivariable regression analysis.

Standardised ordinal logistic regression models were used to evaluate predictors of ETDRS DRSS scores at baseline and at four-years (Table 2; S3 Fig.). VIFs indicated substantial multicollinearity among HbA1c (VIF = 27–38), age (VIF = 19–34), and log-transformed EPO (VIF = 14–34), suggesting potential inflation of standard errors. This is biologically plausible, given the physiological interplay between glycaemic control, ageing, and EPO regulation. In contrast, log-transformed ACR showed minimal collinearity (VIF ≈ 1.1), indicating possible independence from other predictors. Despite the collinearity, the direction and statistical significance of the primary associations remain interpretable and consistent with known pathophysiological mechanisms. At baseline, higher HbA1c (β = 0.994, SE = 0.181, OR = 2.17, *p* < 0.001) and older age (β = 0.064, SE = 0.018, OR = 1.07, *p* = 0.0004) were significantly associated with increased odds of more severe DR. Log-transformed EPO and ACR ratio were not significant predictors.

**Table 2:** Factors associated with baseline and four-year ETDRS DRSS scores.

Predictors of ETDRS			
**Timepoint**	***Predictor***	***OR (95% CI)***	***p-value***
Baseline	Log EPO Baseline	0.55 (0.10 - 3.02)	0.492
	HbA1c Baseline	2.17 (1.45 - 3.04)	<0.001
	Age at enrolment	1.07 (1.03 - 1.11)	<0.001
	Log ACR Baseline	0.70 (0.32 - 1.54)	0.379
Four-years	Log EPO 4YR	0.59 (0.08–4.59)	0.615
	HbA1c 4YR	1.85 (1.30 - 2.63)	<0.001
	Age at enrolment	1.06 (1.02–1.09)	0.002
	Log ACR 4YR	0.72 (0.37–1.41)	0.339

At four-years, HbA1c (β = 0.773, SE = 0.156, OR = 1.85 p < 0.001) and age (β = 0.053, SE = 0.017, OR = 1.05, p = 0.002) remained significant predictors of DR severity, while EPO and ACR continues to show no association. EPO and ACR continued to show no association. Both models demonstrated good fit (baseline: LR χ² = 46.41, *p* < 0.001; four-year: LR χ² = 45.86, *p* < 0.001), explained a substantial portion of variance (Nagelkerke R² = 0.392 and 0.362, respectively), with showed good discrimination (C-index = 0.83 and 0.78).

### Sensitivity analyses for missing data

Of the 112 participants included in the final analysis, 101 (90.2%) had complete baseline data prior to multiple imputation, while 11 (9.8%) required imputation of at least one baseline variable. Sensitivity analyses restricted to participants with complete pre‑imputation data yielded effect estimates that were directionally and interpretively consistent with the primary analyses (S2 and S3 Table). Inclusion of a pre‑imputation completeness indicator in the adjusted models was not independently associated with diabetic retinopathy severity.

## Discussion

This research contributes to the evolving understanding of EPO as a multifaceted molecule in DR, with potential roles extending beyond erythropoiesis into neuroprotection, angiogenesis, and metabolic stress response. Elevated systemic EPO levels observed in individuals with T1DM at both baseline and four-year follow-up suggest that EPO may be upregulated in association with the response to chronic metabolic stress and retinal injury. However, the nature of this elevation could well be adaptive rather than pathogenic [28].

From a physiological perspective, EPO levels might be expected to decline in individuals with worsening DR, particularly as ACR rises, reflecting progressive renal dysfunction [29]. Since the kidney is the primary site of EPO production, progressive diabetic nephropathy is typically associated with reduced EPO synthesis and anaemia [29]. Yet, contrary to this expectation, and in alignment with previous findings amongst individuals with T2DM [30,31], these data show that systemic EPO remains elevated in the T1DM cohort despite higher ACR values. Therefore, EPO elevation in this context may not be driven by renal compensation but may instead reflect extrarenal production and a broader systemic response to tissue hypoxia and inflammation.

This paradox highlights the importance of distinguishing between renal-derived EPO and stress-induced extrarenal EPO. Under hypoxic conditions, the retina is known to express EPO and its receptor EPOR and may upregulate EPO locally as a protective mechanism. In response to tissue injury, EPO may exert this protective effect through a non-EPO-R receptor called the innate repair receptor (IRR), also referred to as the tissue protective receptor (TPR). IRR/TPR is composed of an EPOR monomer and the β common receptor (βCR) [32]. Activation of the IRR/ TPR activates the intracellular signalling pathways JAK2/STAT, PI3K/Akt, and MAPK, thereby reducing inflammation, oedema, and apoptosis [33]. Although EPO binds to this receptor with reduced affinity [34], its upregulation under stress conditions, such as T1DM, is a biologically plausible compensatory mechanism [35]. In the retina, EPO may help mitigate early neurovascular damage, positioning it as a potential therapy target in DR [16,36]. Elevated EPO levels in DR may therefore reflect a response to hypoxia or retinal injury, aimed at preserving neuronal and vascular integrity [19]. Consequently, elevated systemic EPO may serve as a surrogate marker and potential protective factor against retinal cell stress or injury, rather than a reflection of renal function, only contributing to retinal injury via its secondary angiogenic action once neovascularisation commences. It is also important to note that systemic EPO levels are likely to reflect a combination of local retinal production and extrarenal expression from other tissues under metabolic stress, complicating interpretation and reinforcing the need for future studies to explore intraocular EPO dynamics.

Notably, EPO and HbA1c demonstrated substantial collinearity in the multivariate models, which is biologically plausible given their shared relationship with chronic hyperglycaemia and tissue hypoxia [37]. HbA1c reflects cumulative glycaemic burden, while EPO may be upregulated in response to the resulting metabolic stress. This physiological linkage likely contributes to the statistical collinearity observed, and although it may inflate the standard errors, the direction and significance of the association remain interpretable, reinforcing the relevance of both markers in the context of diabetic retinopathy [30].

Despite the known neuroprotective and anti-inflammatory roles of EPO, the present findings show no association between EPO and corneal nerve integrity as measured by the CCM parameters CNFD, CNBD, and CNFL. This disconnect suggest that EPOs protective effects may be tissue-specific, more relevant to the retinal neurons than to the peripheral nerves, or that its protective actions are functional rather than structural, modulating inflammation or apoptosis without necessarily preserving measurable nerve morphology. This may be particularly true if EPOs role is more anti-apoptotic [38,39] or anti-inflammatory [40,41] than regenerative.

In alignment with the hypothesis that EPO acts as a tissue-protective factor [16,32], the observed associations between EPO and ETDRS DRSS scores may reflect ongoing retinal stress or a compensatory attempt at tissue preservation, rather than a direct causal contribution to disease progression. Regression analyses revealed that both baseline and four-year EPO levels were significantly associated with ETDRS DRSS scores at four-years, indicating a consistent relationship between elevated EPO and greater DR severity. However, in multivariate models, HbA1c and age emerged as the dominant predictors of DR severity, while EPO and ACR were not independently associated. This underscores the multifactorial nature of DR progression and suggests that EPO, while biologically relevant, may not be a strong standalone predictor of disease severity. Instead, its elevation may be part of a broader adaptive response to retinal injury and metabolic stress [42], potentially reflecting extrarenal EPO expression in response to hypoxia and inflammation. These findings support the interpretation of systemic EPO and as surrogate marker or retinal stress, rather than direct driver of early DR progression [30].

A key consideration in interpreting the relationship between systemic EPO levels and DR severity is confounding by diabetic neurodegeneration. Reduced EPO levels have been reported in diabetic neuropathy, and corneal nerve fibre loss reflects broader neurodegenerative burden. As corneal confocal microscopy–derived metrics may lie on a shared neurodegenerative pathway rather than act as independent confounders, these measures were not included in the primary multivariable models assessing ETDRS retinopathy severity. Consistent with this interpretation, EPO was not independently associated with retinopathy severity after adjustment for age and glycaemic exposure.

Importantly, the observed associations between systemic EPO and DR severity were modest in magnitude. Although ordinal regression models demonstrated statistical associations with follow-up severity, the present analyses were not designed to evaluate clinical predictive performance, and no measures of discrimination, calibration, or clinical utility were assessed. These findings should therefore be interpreted as identifying an exploratory biomarker signal rather than a validated prediction model.

This longitudinal analysis supports the potential of systemic EPO as a predictor of DR severity, addressing a key gap in prior cross-sectional research. Taken together, these findings reinforce emerging perspectives on EPO as a multifaceted molecule in DR, not solely a haematopoietic hormone, but also a tissue-protective agent [32,33]. The elevation of EPO observed in DR may therefore represent a compensatory response to retinal stress early in the disease rather than a direct pathogenic driver. However, the lack of predictive value in adjusted models suggests that EPO may be best interpreted as part of a broad biomarker panel, rather than a singular indicator of disease progression. This nuance is critical in understanding whether systemic EPO reflects established retinal damage or serves as an early signal of disease activity, an unresolved question that underscores the need for further longitudinal research.

An additional limitation relates to the modelling of DR severity across individual ETDRS stages. Although estimating stage‑specific odds could provide insight into whether particular severity transitions disproportionately influence observed associations, this was not feasible in the present study due to limited sample sizes within individual ETDRS categories. To avoid unstable or misleading estimates, retinopathy severity was therefore modelled as an ordinal outcome reflecting cumulative disease burden across the ETDRS scale rather than discrete stage transitions. Larger studies with sufficient representation across severity strata will be required to examine potential stage‑specific effects.

Future studies may contribute significantly by exploring intraocular EPO concentrations, which may more directly reflect retinal pathology, and investigate whether EPO dynamics differ across DR phenotypes, stages or treatment responses. Additionally, the limited sample size in this study constrained the ability to stratify by renal function or to include additional covariates without risking model overfitting. Given the biological relevance of renal function to systemic EPO levels, its influence could not fully be disentangled. Larger cohorts may be better positioned to explore the interplay between renal and retinal sources of EPO and their respective contributions to DR severity and progression.

## Supporting information

S1 Fig Outcomes of CCM assessment. Mann-Whitney U tests indicate a significantly lower density of nerve fibres in the cornea (CNFD) in the diabetes group than the control group at both baseline (U = 993.50, Z = −3.513, P < 0.001) and 4-years (U = 959.00, Z = −3.705, p < 0.001); significantly lower corneal nerve branch density (CNBD) at baseline (U = 931.00, Z = −3.861, P < 0.001) and 4-years (U = 974.50, Z = −3.617, p < 0.001); significantly lower corneal fibre length (CNFL) at baseline (U = 868.00, Z = −4.213, P < 0.001) and 4-years (U = 994.50, Z = −3.784, p < 0.001).
(TIF)

S2 Fig Baseline ETDRS DRSS. Mann-Whitney U tests indicate a significantly higher ETDRS DRSS score in the diabetes group than the control group at both baseline (U = 767.00, Z = −5.205, P < 0.001) and 4-years (U = 764.50, Z = −4.929, P < 0.001). Changes in ETDRS DRSS scores over time were assessed for both the diabetes and control groups. Individuals with diabetes saw a significant increase in ETDRS DRSS scores from baseline to 4-years as indicated by Wilcoxon Signed-Rank Test (z = −4.495, P < 0.001), indicating that the diabetic group experienced worsening DR over the 4-year period. This trend was not apparent in the control group (z = −1.701, P = 0.089), suggesting that the control group’s macular function remained stable. Six participants with T1DM had missing ETDRS DRSS scores and were excluded from this analysis.
(TIF)

S3 Fig ETDRS DRSS Predictors. Forest plot of predictors of ETDRS DRSS score at baseline and four-years. Odds ratios with 95% confidence intervals are shown for each predictor. Higher HbA1c and older age at enrolment were significantly associated with increased DR severity at both timepoints. Log-transformed EPO and ACR were not significantly associated with DR outcomes. The confidence intervals for EPO are notably wide at both baseline and four-years, reflecting statistical uncertainty likely driven by high collinearity with HbA1c (VIF > 20), relatively small effect sizes, and sparse data in some ETDRS categories. These factors may reduce model stability and inflate the standard errors, suggesting that EPO contributes little additional predictive value once HbA1c is included in the model.
(TIF)

S1 Table Censored value conversions. To ensure consistency and enable statistical analysis, the following adjustments and assumptions were made during data preparation. These decisions align with the *Ethical Guidelines for Statistical Practice* and were applied uniformly across the dataset. Certain laboratory results reported as greater than (>) or less than (<) specific values were converted to numeric values for statistical analysis. These substitutions were made to allow inclusion in quantitative analysis while minimising distortion of variance. For example, replacing “ > 90” with 90 for eGFR may slightly underestimate true renal function in some individuals, but this conservative estimate allows for consistent statistical treatment. These approaches are commonly accepted when values exceed or fall below quantifiable limits. In alignment with the principles outlined in the ethical guidelines for statistical practice, use of conservative substitution reflects an appropriate method for maintain dataset completely and analytic consistency [43].
(DOCX)

S2 Table Missing data summary. To address missing data and reduce bias, 10-fold multiple imputation was employed across the dataset. Missing counts represent unavailable values in the original LANDMark dataset prior to multiple imputation. Percentages are calculated relative to group‑specific baseline sample sizes (45 controls and 72 participants with T1DM).
(DOCX)

S3 Table Sensitivity analysis. Sensitivity analysis assessing robustness of ordinal regression results to missing data handling. MI = multiple imputation; OR = odds ratio; CI = confidence interval. Complete‑case analyses were restricted to participants with complete baseline data prior to multiple imputation. Threshold‑excluded analyses removed observations with values recoded at assay detection or reporting limits. The pre‑imputation completeness indicator was not independently associated with diabetic retinopathy severity. † Sample size reflects exclusion of participants with threshold‑affected values in the threshold‑excluded sensitivity analysis.
(DOCX)
