# Supplementary material for: Exploring systemic erythropoietin in diabetic retinopathy: Longitudinal associations and biomarker potential in the LANDMark cohort
Source: PLoS One. 2026 Sep 15;21(9):e0358455. doi: 10.1371/journal.pone.0358455 (PMC13577516; doi:10.1371/journal.pone.0358455)
Supplement: Supplementary file 1 [file pone.0358455.s004.docx]

***S1 Table:*** *Censored value conversions*

| **Parameter** | **Reported value** | **Converted value** |
| --- | --- | --- |
| eGFR (mL/min) | >90 | 90 |
| Albumin (g/L) | <2.0 | 2.0 |
| Urine ACR (mg/mmol) | <2.0 | 2.0 |
|  | <4.0 | 4.0 |
|  | <1.3 | 1.3 |
|  | <0.6 | 0.6 |
|  | <0.2 | 0.2 |
| eGFR, estimated glomerular filtration rate; ACR, albumin-creatinine ratio | | |
