# Supplementary material for: Exploring systemic erythropoietin in diabetic retinopathy: Longitudinal associations and biomarker potential in the LANDMark cohort
Source: PLoS One. 2026 Sep 15;21(9):e0358455. doi: 10.1371/journal.pone.0358455 (PMC13577516; doi:10.1371/journal.pone.0358455)
Supplement: Supplementary file 2 [file pone.0358455.s005.docx]

**S2 Table:** Missing data summary

| **Section** | **Group** | **Variable** | **Missing count (n, % of group)** |
| --- | --- | --- | --- |
| B.1 | Baseline (Control) | Urine albumin (mg/L) | 3 (6.7%) |
| B.1 | Baseline (Control) | Urine albumin/creatinine ratio (mg/mmol) | 2 (4.4%) |
| B.1 | Baseline (Control) | Creatinine (µmol/L) | 1 (2.2%) |
| B.1 | Baseline (Control) | eGFR (mL/min) | 1 (2.2%) |
| B.1 | Baseline (Control) | LDL Cholesterol (mmol/L) | 1 (2.2%) |
| B.1 | Baseline (Control) | QST vibration threshold average (Hz) | 1 (2.2%) |
| B.1 | Baseline (Control) | Waist circumference (average) | 3 (6.7%) |
| B.2 | 4‑Year (Control) | Urine albumin (mg/L) | 1 (2.2%) |
| B.2 | 4‑Year (Control) | Urine albumin/creatinine ratio (mg/mmol) | 1 (2.2%) |
| B.2 | 4‑Year (Control) | QST vibration threshold average (Hz) | 3 (6.7%) |
| B.2 | 4‑Year (Control) | Erythropoietin (EPO) | 2 (4.4%) |
| B.3 | Baseline (T1DM) | Erythropoietin (EPO) | 5 (6.9%) |
| B.3 | Baseline (T1DM) | HbA1c | 2 (2.8%) |
| B.3 | Baseline (T1DM) | Urine albumin (mg/L) | 3 (4.2%) |
| B.3 | Baseline (T1DM) | Urine albumin/creatinine ratio (mg/mmol) | 5 (6.9%) |
| B.3 | Baseline (T1DM) | Creatinine (µmol/L) | 2 (2.8%) |
| B.3 | Baseline (T1DM) | eGFR (mL/min) | 6 (8.3%) |
| B.3 | Baseline (T1DM) | LDL Cholesterol (mmol/L) | 1 (1.4%) |
| B.3 | Baseline (T1DM) | QST vibration threshold average (Hz) | 1 (1.4%) |
| B.3 | Baseline (T1DM) | Waist circumference (average) | 6 (8.3%) |
| B.3 | Baseline (T1DM) | Neuropathy Disability Score (NDS) | 1 (1.4%) |
| B.3 | Baseline (T1DM) | Peroneal CV (ankle to fibular head, m/s) | 6 (8.3%) |
| B.4 | 4‑Year (T1DM) | Urine albumin (mg/L) | 2 (2.8%) |
| B.4 | 4‑Year (T1DM) | Urine albumin/creatinine ratio (ACR) | 2 (2.8%) |
| B.4 | 4‑Year (T1DM) | eGFR (mL/min) | 2 (2.8%) |
| B.4 | 4‑Year (T1DM) | QST cold sensation threshold average (°C) | 2 (2.8%) |
| B.4 | 4‑Year (T1DM) | QST warm sensation threshold average (°C) | 2 (2.8%) |
| B.4 | 4‑Year (T1DM) | QST vibration threshold average (Hz) | 5 (6.9%) |
| B.4 | 4‑Year (T1DM) | Neuropathy Disability Score (NDS) | 1 (1.4%) |
| B.4 | 4‑Year (T1DM) | Peroneal CV (ankle to fibular head, m/s) | 7 (9.7%) |
