# Supplementary material for: Exploring systemic erythropoietin in diabetic retinopathy: Longitudinal associations and biomarker potential in the LANDMark cohort
Source: PLoS One. 2026 Sep 15;21(9):e0358455. doi: 10.1371/journal.pone.0358455 (PMC13577516; doi:10.1371/journal.pone.0358455)
Supplement: Supplementary file 3 [file pone.0358455.s006.docx]

***Table B2.*** *Sensitivity analyses assessing robustness of ordinal regression results to missing data handling*

| **Analysis** | **Sample included** | **n** | **Baseline EPO [OR, 95% CI]** | **HbA1c**  **[OR, 95% CI]** | **Age**  **[OR, 95% CI]** | **ACR**  **[OR, 95% CI]** |
| --- | --- | --- | --- | --- | --- | --- |
| Primary model (MI) | All participants (imputed dataset) | 112 | **1.98 (0.39–10.05)** | **2.06 (1.53–2.77)** | **1.05 (1.02–1.09)** | **0.71 (0.37–1.39)** |
| Complete‑case sensitivity analysis | Participants with complete baseline data prior to MI | 101 | **1.61 (0.28–9.12)** | **2.02 (1.48–2.76)** | **1.04 (1.01–1.08)** | **0.64 (0.30–1.34)** |
| Threshold‑excluded sensitivity analysis | Threshold‑affected values excluded | 101† | Effect estimates unchanged from primary model | Effect estimates unchanged | Effect estimates unchanged | Effect estimates unchanged |
| Imputation‑indicator model | All participants PLUS pre‑MI completeness indicator | 112 | **1.99 (0.39–10.13)** | **2.04 (1.52–2.75)** | **1.05 (1.02–1.08)** | **0.69 (0.35–1.38)** |
